# Detoxification Role of Metabolic Glutathione S-Transferase (GST) Genes in Blood Lead Concentrations of Jamaican Children with and without Autism Spectrum Disorder

**DOI:** 10.3390/genes13060975

**Published:** 2022-05-29

**Authors:** Mohammad H. Rahbar, Maureen Samms-Vaughan, Sori Kim, Sepideh Saroukhani, Jan Bressler, Manouchehr Hessabi, Megan L. Grove, Sydonnie Shakspeare-Pellington, Katherine A. Loveland

**Affiliations:** 1 Department of Epidemiology, Human Genetics, and Environmental Sciences (EHGES), School of Public Health, The University of Texas Health Science Center at Houston, Houston, TX 77030, USA; jan.bressler@uth.tmc.edu (J.B.); megan.l.grove@uth.tmc.edu (M.L.G.); 2 Biostatistics/Epidemiology/Research Design (BERD) Component, Center for Clinical and Translational Sciences (CCTS), The University of Texas Health Science Center at Houston, Houston, TX 77030, USA; sori.kim@uth.tmc.edu (S.K.); sepideh.saroukhani@uth.tmc.edu (S.S.); manouchehr.hessabi@uth.tmc.edu (M.H.); 3 Division of Clinical and Translational Sciences, Department of Internal Medicine, McGovern Medical School, The University of Texas Health Science Center at Houston, Houston, TX 77030, USA; 4 Department of Child & Adolescent Health, The University of the West Indies (UWI), Mona Campus, Kingston 7, Jamaica; msammsvaughan@gmail.com (M.S.-V.); sydonniesp@gmail.com (S.S.-P.); 5 Department of Biostatistics & Data Science, School of Public Health, The University of Texas Health Science Center at Houston, Houston, TX 77030, USA; 6 Human Genetics Center, School of Public Health, The University of Texas Health Science Center at Houston, Houston, TX 77030, USA; 7 Louis A Faillace, MD, Department of Psychiatry and Behavioral Sciences, McGovern Medical School, The University of Texas Health Science Center at Houston, Houston, TX 77054, USA; katherine.a.loveland@uth.tmc.edu

**Keywords:** heavy metals, blood lead concentrations, glutathione S-transferase (GST) genes, detoxification, autism spectrum disorder (ASD), Jamaica

## Abstract

Glutathione S-transferases (GST) are involved in the detoxification of exogenous chemicals including lead (Pb). Using data from 344 pairs of autism spectrum disorder (ASD) cases and age- and sex-matched typically developing (TD) controls (2–8 years old) from Jamaica, we investigated the interaction between three GST genes and ASD status as determinants of blood Pb concentrations (BPbCs). We found that ASD cases had lower geometric mean BPbCs than TD children (1.74 vs. 2.27 µg/dL, *p* < 0.01). Using a co-dominant genetic model, ASD cases with the Ile/Val genotype for the *GSTP1* Ile105Val polymorphism had lower GM BPbCs than TD controls, after adjusting for a known interaction between *GSTP1* and *GSTT1*, child’s parish, socioeconomic status, consumption of lettuce, fried plantains, and canned fish (Ile/Val: 1.78 vs. 2.13 µg/dL, *p* = 0.03). Similarly, among carriers of the I/I or I/D (I*) genotype for *GSTT1* and *GSTM1*, ASD cases had lower adjusted GM BPbCs than TD controls (*GSTT1* I*: 1.61 vs. 1.91 µg/dL, *p* = 0.01; *GSTM1* I*: 1.71 vs. 2.04 µg/dL, *p* = 0.01). Our findings suggest that genetic polymorphisms in GST genes may influence detoxification of Pb by the enzymes they encode in Jamaican children with and without ASD.

## 1. Introduction

Lead (Pb) is a toxic metal that has deleterious effects on the human body and has been recognized as a major public health risk, particularly in developing countries [1,2,3]. Pb poisoning has been reported as a factor associated with neurodevelopmental impairment during childhood [4]. Blood Pb concentrations (BPbCs) ≥ 5 μg/dL were defined as “level of concern” by the US Centers for Disease Control and Prevention (CDC) [5], with no Pb level currently considered safe [6,7,8].

Some but not all studies have previously found associations between environmental exposure to Pb and autism spectrum disorder (ASD). For example, an age-matched case–control study of 40 pairs of boys 4–8 years old from Kuwait reported that ASD cases had a significantly higher level of Pb in the hair than TD controls ((median Pb levels in ASD cases: 6.75 μg/g) vs. (median Pb levels in TD controls: 3.20 μg/g), *p* < 0.01) [9]. A recent study of 52 ASD cases and 30 TD controls (3–12 years old) from the Middle East found that mean red blood cell Pb concentrations were significantly lower for the TD children than for the children with ASD (4.73 μg/dL vs. 6.79 μg/dL, *p* < 0.01) [10]. Another age- and sex-matched study, from India, also showed that children with ASD and different levels of functioning had significantly higher Pb levels in hair samples (*p* < 0.01) than TD controls [11]. Furthermore, a positive association was observed between hair levels of Pb and both verbal communications and general impression severity scores in children with ASD from Saudi Arabia (*p* = 0.02 and *p* < 0.01, respectively) [12]. In contrast, the investigators in another study that reported significantly lower urine Pb concentrations in children with ASD than in TD controls (1.19 μg/g creatinine vs. 4.63 μg/g creatinine, *p* < 0.01) suggested that this could be due to a decreased ability to detoxify heavy metals [13]. On the other hand, a study of 2–5-year-old children in California enrolled in Childhood Autism Risk from Genetics and Environment (CHARGE) that compared mean BPbCs between 37 ASD cases and 15 TD controls did not show any significant differences (*p* = 0.97) [14]. Notably, most of the aforementioned studies did not adjust their findings to control for potential confounding variables such as food consumed and other environmental exposures. However, our results from the Epidemiological Research on Autism in Jamaica (ERAJ) study in Jamaica showed an inverse association of Pb with ASD from univariable general linear models (GLM) and quantile regression models as well as a weighted quantile sum regression (WQS) mixture index score with ASD, though when adjusted for potential confounding variables including diet, this association was no longer statistically significant. This finding could be due to some children with ASD eating a more restricted diet compared to TD children, possibly due to the higher rate of gastrointestinal symptoms and sensory sensitivities [15].

In addition to the need for adjusting for the potential confounding role of dietary intake and other potential confounding variables while assessing associations between BPbCs and ASD, several studies have also suggested that differences in Pb concentrations could be due to varied detoxification and excretory mechanisms in children with and without ASD [10,16,17]. It has also been shown that Pb-induced toxicity is associated with chronic oxidative stress and mitochondrial dysfunction [18,19,20,21]. 

Glutathione S-transferases (GST) are a family of enzymes that play a key role in the detoxification of xenobiotics, including heavy metals such as Pb, by catalyzing their conjugation with reduced glutathione (GSH), and also by protecting cells against oxidative stress [22,23,24,25]. GST genes (e.g., *GSTP1*, *GSTT1*, and *GSTM1)* are highly polymorphic metabolic genes that encode GST enzymes [23]. Polymorphisms in these genes may influence their biological function and detoxification capacity. For example, null alleles of *GSTT1* and *GSTM1* can completely interrupt the enzymes’ function [26]. There are also findings suggesting that certain GST polymorphisms may influence the individuals’ susceptibility to the cytotoxic effects of Pb [27,28,29]. In addition, dysfunctions in the glutathione and mitochondrial systems, as well as the presence of chronic oxidative stress in the brains and blood of children with ASD, have been reported previously [30,31,32,33,34,35,36]. There is also evidence of associations between GST gene polymorphisms and ASD. For example, findings of a family-based association study showed significant over-transmission of a haplotype with two polymorphisms in *GSTP1* (Ala114Val and Ile105Val) in the mothers of children with ASD [37]. Other case–control studies reported a significant association between the *GSTM1* null polymorphism and about two times higher odds of ASD [36,38]. We have also previously reported a significant interaction between *GSTT1* and *GSTP1* in relation to ASD indicating that Jamaican children with ASD who are heterozygous for the *GSTP1* Ile105Val polymorphism have nearly three times higher odds of also carrying the *GSTT1* D/D genotype than TD controls when using a co-dominant genetic model for *GSTP1* [39]. These studies seem to indicate that genetic variation may convey varied Pb-induced susceptibility to neurodevelopmental disorders such as ASD. However, research on this topic has been very limited so far.

We have carried out a long-term collaborative study to assess the role of additive and interactive associations of environmental exposures to six metals and three GST genes (*GSTP1*, *GSTM1*, and *GSTT1*) in the development of ASD in Jamaican children. Although our previous study did not find any significant interactions between BPbCs and genotypes for any of the aforementioned three GST genes in relation to ASD status, using different conditional logistic regression (CLR) models, (all *p* for interaction between BPbCs and each of the GST genes > 0.49), a marginally significant interactive association of *GSTP1* with a mixture of Pb, Hg, and Mn was detected (*p* = 0.07) based on a negative association generalized weighted quantile sum (gWQS) model [40]. From another ERAJ study, we also reported significant interactions between *GSTP1* and ASD status in relation to blood arsenic (*p* = 0.04) [41] and blood mercury (*p* < 0.01) [42], as well as blood aluminum concentrations (*p* < 0.02) [43]. Based on the accumulation of evidence for the complex role of metabolic genes in detoxification of heavy metals in relation to ASD, we investigated the possible interaction of each of the three GST genes with ASD status and possible pairwise gene–gene interactions in relation to BPbCs of Jamaican children.

## 2. Materials and Methods

### 2.1. Epidemiological Research on Autism in Jamaica (ERAJ) Studies 

ERAJ and ERAJ-Phase 2 (ERAJ-2) are 1:1 age- and sex-matched case–control studies of Jamaican children 2–8 years old with ASD and their TD controls that investigated potential individual or interactive associations between environmental exposures and three GST genes (*GSTP1*, *GSTT1*, and *GSTM1*) in relation to ASD as previously described [44]. The Autism Diagnostic Observation Schedule, Second Edition (ADOS-2) [45] and Autism Diagnostic Interview-Revised (ADI-R) [46] were administered to determine ASD status for children included in the Jamaican Autism Database. The absence of developmental disorders in the TD control children was determined based on a score < 6 for the Social Communication Questionnaire (SCQ) [47].

We also elicited responses to a socioeconomic status (SES) questionnaire and to a food frequency questionnaire [48], and collected 4–5 mL of whole blood from each child for assessing exposure to lead and some other metals and for use in the genotyping assays.

Here, we used data from 344 ASD cases, and age- (±6 months) and sex-matched TD controls (n = 688 that includes 344 matched pairs), enrolled in the ERAJ studies based on the study protocol that was approved by the Institutional Review Boards (IRBs) of UTHealth, UWI, and Michigan Department of Health and Human Services (MDHHS), in Lansing, Michigan. Written informed consent was obtained from parents and assent from the children when applicable (for details, please refer to the section on “Institutional Review Board Statement”).

### 2.2. Assessment of Pb Exposure

A 2 mL aliquot of the 4–5 mL sample of whole venous blood collected after assessment of children for ASD or TD control status was used to measure BPbCs. Details regarding the processing and storage of samples at the Caribbean Genetics (CARIGEN) lab at UWI, their shipment to MDHHS, and their analysis and quality control (QC) at MDHHS have been reported previously [49]. Two different limits of detection (LoD) were established by MDHHS for Pb during the two successive phases of the ERAJ study due to changes in technology (0.25 ug/dL in ERAJ and 0.30 ug/dL in ERAJ-2). However, in both phases of the ERAJ studies, all of the BPbCs were above their respective LoDs. 

### 2.3. Genetic Analysis

In this study, we analyzed variants in three different GST genes and their possible associations with ASD, as well as interactive associations between genotypes for the three genes and ASD status in relation to BPbCs. These variants included the *GSTP1* Ile105Val polymorphism (rs1695) and insertion/deletion polymorphisms in both *GSTT1* and *GSTM1*. Methods for DNA extraction and genotyping have been described previously [15,39,42]. There are three genotypes for the *GSTP1* polymorphism (Ile/Ile, Ile/Val, Val/Val) and two for *GSTT1* and *GSTM1* (D/D and I*) since I/I and I/D cannot be distinguished. Three genetic models for *GSTP1* (dominant, co-dominant, and recessive) and one genetic model for *GSTT1* and *GSTM1* (recessive) were tested.

### 2.4. Statistical Analysis

We reported the geometric mean (GM) after using the natural logarithm (ln) to transform the BPbCs due to their skewed distribution. Using conditional logistic regression (CLR) models, we compared ASD cases and TD control groups with respect to various characteristics of children and their parents, including the distributions of the children’s *GSTP1*, *GSTM1*, and *GSTTI* genotypes and their dietary or environmental exposures. Using univariable general linear models (GLMs) in which the log-transformed BPbCs were the dependent variable, we also assessed the possible association of each of the three GST genes with ASD status, sociodemographic characteristics, and consumption of various types of food. In order to account for the potential clustering effect of 344 matched pairs in all GLMs, we entered 343 dummy variables as described previously [42].

We also used multivariable GLMs to explore the possible interactive associations between the genotypes for each of the three GST genes and ASD status in relation to BPbCs. In addition, in adjusted multivariable GLMs, we included the parish in which the child was born, car ownership by the family as an index of SES, and consumption of lettuce, fried plantains, and sardine or mackerel fish, which have previously been identified as having significant associations with ASD status and BPbCs [15,50]. In order to minimize potential effects of multicollinearity, we only retained one of any two correlated variables after initially checking pairwise correlations between all pairs of individual covariates and finding that the model became unstable when both were added. We assessed two-way interactions between genotypes of the three GST genes in relation to BPbCs using multivariable GLMs, and also accounted for a previously reported interaction between *GSTP1* and *GSTT1* when the adjusted models included these two genes. We used the CONTRAST statement in PROC GLM in SAS [51] to assess whether the differences between GM BPbCs found when comparing ASD cases and TD controls were significantly different between/among those with different genotypes for the three GST genes. Similarly, we tested whether there was a significant difference in the GM BPbCs between/among ASD cases and TD controls by GST genotype. Unadjusted and adjusted GM BPbCs were calculated for both ASD cases and TD controls with different GST genotypes. A statistical threshold of p < 0.05 was used to determine statistical significance for all statistical tests without accounting for multiple comparisons. We utilized SAS 9.4 software for all analyses [52].

## 3. Results

At enrollment, 71.8% of ASD cases and 74.7% of the TD controls were 48 months or older. Almost all of the participants in this study were Afro-Caribbean (94.5% of ASD cases, 97.1% of TD control children, as well as over 95% of their parents). Higher proportions of both parents of ASD cases were older than those of the TD controls (19.5% of the mothers and 43.8% of fathers of ASD cases were 35 years or older vs. 11.8% of mothers and 30.3% of fathers of TD controls, all *p* < 0.01) when their children were born. Similarly, 62.3% of ASD cases and 48.3% of TD controls had at least one parent who had attained postsecondary education (*p* < 0.01). A larger proportion of ASD cases were from families with higher SES compared to TD controls, with 54.1% of ASD case families owning a car versus 41.9% of car ownership among TD control families (*p* < 0.01). There was no significant difference in the frequencies of *GSTP1*, *GSTM1*, and *GSTT1* genotypes between ASD cases and TD controls (all *p* > 0.07). Among TD children, 24.0% and 25.9% had the null genotype (DD) for *GSTM1* and *GSTT1*, respectively. The arithmetic mean BPbC was 2.41µg/dL for ASD cases and 2.94 µg/dL for TD controls (*p* < 0.01). Details regarding characteristics of children and their parents are provided in Table 1.

Comparison of the consumption of various food items between ASD case and TD control children revealed that compared to TD control children, ASD cases consumed lower levels of root vegetables (yam, sweet potato, or dasheen (matched odds ratio (MOR) (95% CI) = 0.59 (0.43, 0.82), *p* < 0.01) and carrot or pumpkin (MOR = 0.44, (0.30, 0.67), *p* < 0.01). Similar comparisons of the distributions of other dietary and environmental factors are displayed in Table 2. 

In Table 3, we reported on potential associations of ASD status and various sociodemographic and dietary exposures, as well as children’s genotype for GST genes with BPbCs based on univariable GLMs. We found a significant association between ASD status and BPbCs (GM BPbC for ASD group = 1.74 µg/dL vs. 2.27 µg/dL for the TD control group, *p* < 0.01). We also found that child’s age at enrollment, place of child’s birth (parish), and SES were significantly associated with BPbCs (all *p* ≤ 0.02). Regarding dietary factors, we found that consumption of root vegetables, namely yam, sweet potato or dasheen, and carrot or pumpkin, as well as some types of fruits including tomatoes, ackee, avocado, and fried plantains were significantly associated with BPbCs (all *p* < 0.03). Similarly, we found a significantly higher GM BPbC among children who had higher seafood consumption (more than 6 meals per week), consumption of fresh water fish (pond fish and tilapia), canned fish (sardine and mackerel), and salted fish and shellfish (all *p* < 0.02). The associations between other independent variables and BPbCs were marginally significant (callaloo, broccoli, or pak choi (*p* = 0.07); green banana (*p* = 0.05); saltwater fish (*p* = 0.05)). There were no significant associations between any of the GST genes and BPbCs (*GSTT1* I* (I/I or I/D): *p* = 0.52; *GSTM1* I* (I/I or I/D): *p* = 0.40; *GSTP1*: *p* > 0.77 for all pairwise comparisons).

In multivariable GLMs, we did not find any significant interactions between GST genes and ASD status in relation to BPbCs, either in the unadjusted model, or after accounting for the interaction between *GSTT1* and *GSTP1* and further adjustment for place of child’s birth (parish), SES (car ownership by the family), consumption of lettuce, fried plantains, and sardine or mackerel fish (all overall interaction *p* > 0.5). For example, using a co-dominant genetic model for *GSTP1* and after adjusting for the aforementioned variables, although not statistically significant, we found that while ASD cases with Ile/Ile genotype had lower GM BPbC than those with Ile/Val genotype (1.55 µg/dL vs. 1.78 µg/dL, *p* = 0.25), ASD cases with either Ile/Ile or Ile/Val genotype had a higher GM BPbC than those with Val/Val genotype (GM BPbC among ASD cases with Ile/Ile vs. Val/Val genotype for *GSTP1*: 1.55 µg/dL vs. 1.50 µg/dL, *p* = 0.82; GM BPbC among ASD cases with Ile/Val vs. Val/Val genotype for *GSTP1*: 1.78 µg/dL vs. 1.50 µg/dL, *p* = 0.19). Similar non-significant differences were observed among TD children with various genotypes for *GSTP1* using the co-dominant genetic model (all *p* ≥ 0.16). Similarly, using the recessive model for *GSTT1*, although the differences were not statistically significant, we found that TD children with I/I or I/D genotype had lower BPbCs than those with DD genotype (1.91 µg/dL vs. 1.99 µg/dL, *p* = 0.71), whereas ASD cases with I/I or I/D genotype had higher GM BPbC than those with DD genotype (1.61 µg/dL vs. 1.60 µg/dL, *p* = 0.97). Additional details about the comparisons of GM BPbC among ASD cases and TD control children with various genotypes for GST genes are provided in Table 4. 

Although the interaction between genotype for GST genes and ASD status was not significant in relation to BPbCs, ASD cases with certain genotypes for GST genes had significantly lower GM BPbC than TD control children with the same genotype for GST genes. Specifically, using the co-dominant model for *GSTP1*, while there was no significant association between ASD status and BPbCs among children with Ile/Ile genotype in both unadjusted and adjusted models, ASD cases with Ile/Val genotype had significantly lower GM BPbC than TD control children with the same genotype for *GSTP1*, in both unadjusted and adjusted models (unadjusted GM: 1.78 µg/dL vs. 2.31 µg/dL, *p* < 0.01; adjusted: 1.78 µg/dL vs. 2.13 µg/dL, *p* = 0.03). Similarly, using the co-dominant model for *GSTP1*, ASD cases with Val/Val genotype also had significantly lower GM BPbC than TD control children with the same genotype for *GSTP1* in the unadjusted model (1.68 µg/dL vs. 2.30 µg/dL, *p* = 0.02); however, the difference became marginally significant after adjusting for the aforementioned covariates (1.50 µg/dL vs. 1.94 µg/dL, *p* = 0.06).

In similar analyses for *GSTT1*, we did not find any significant interaction between *GSTT1* and ASD status in relation to BPbCs before and after adjusting for the aforementioned covariates (*p* = 0.73 and *p* = 0.77, respectively). However, we found a significant difference in the unadjusted and adjusted GM BPbC between ASD cases and TD control children with I/I or I/D genotype for *GSTT1* (unadjusted GM: 1.72 µg/dL vs. 2.26 µg/dL, *p* < 0.01; adjusted GM: 1.61 µg/dL vs. 1.91 µg/dL, *p* = 0.01). The difference between unadjusted and adjusted GM BPbC for ASD cases and TD control children with DD genotype was marginally significant (both *p* = 0.09). Details for the difference in GM BPbC between ASD cases and TD control children by *GSTT1* genotypes are displayed in Table 5.

There were no significant interactive associations between *GSTM1* and ASD status in relation to BPbCs before and after adjusting for parish at child’s birth, SES (car ownership by the family), and consumption of lettuce, fried plantains, and sardine or mackerel fish (unadjusted: *p* = 0.57, adjusted: *p* = 0.82). However, in the unadjusted model, we found that ASD cases with the *GSTM1* D/D genotype had a significantly lower GM BPbC than TD controls with the same genotype (1.67 µg/dL vs. 2.31 µg/dL, *p* = 0.01), although this difference was not significant after adjusting for the aforementioned covariates (1.58 µg/dL vs. 1.81 µg/dL, *p* = 0.28). Among those with the *GSTM1* I/I or I/D genotype, ASD cases had significantly lower GM BPbC than TD controls before and after adjusting for the same covariates (unadjusted GM: 1.78 µg/dL vs. 2.26 µg/dL, *p* < 0.01; adjusted GM: 1.71 µg/dL vs. 2.04 µg/dL, *p* = 0.01). Details for the difference in GM of BPbC between ASD cases and TD controls by *GSTM1* genotypes are shown in Table 5.

## 4. Discussion

To our knowledge, this is the first study to investigate whether the associations between BPbCs and GST genes depend on ASD status in 2–8-year-old Jamaican children by assessing possible interactions of *GSTP1, GSTT1 and GSTM1* and ASD status in relation to BPbCs. Although we did not find any statistically significant interactions between any of the three GST genes and ASD status in relation to BPbCs, overall, ASD children tended to have lower BPbCs than TD children, and the GM difference in BPbCs between ASD cases and TD controls was either significant or marginally significant only among children with certain genotypes for GST genes.

For example, using either a co-dominant or dominant genetic model for *GSTP1*, our findings suggest that in the presence of at least one *GSTP1* Val105 allele, children with ASD had significantly lower GM BPbC than TD children, before and after adjusting for the *GSTT1* and *GSTP1* interaction, as well as SES, place of child’s birth (parish), and consumption of fried plantains, lettuce, and canned fish. Similarly, even though there were no significant interactions found between either the *GSTT1* or *GSTM1* genes and ASD status in relation to BPbCs, our findings from the adjusted model suggested that GM BPbCs were significantly lower in children with ASD than in TD children only among those with I* genotypes for either *GSTT1* or *GSTM1*. 

The inverse associations between BPbCs and ASD status that we found in our additive models are consistent with previous studies that assessed Pb concentrations in hair, urine and blood samples of children with and without ASD in different regions of the world. For example, findings from a case–control study of 354 ASD cases and 241 TD children (0–15 years old) in Japan reported a significantly lower Pb level in hair samples from ASD cases than from TD controls (mean (SD) = 2.52 (0.37) parts per billion (ppb) for children with ASD vs. 2.89 (0.34) ppb for TD controls, *p* < 0.01) only among male participants. [54]. Though marginally significant, an age- and sex-matched case–control study of 2–9 year-old children (74 ASD cases vs. 74 TD controls) in Russia also reported ASD cases had a lower mean hair Pb level than TD controls (mean (SD) (95% CI) for ASD = 0.45 μg/g (0.21, 0.61) vs. TD = 0.59 μg/g (0.30, 1.05), *p* = 0.06) [55]. Another study, from Turkey, that compared 30 ASD cases and 20 TD control children (3–12 years old) reported a significantly lower urine Pb concentration in children with ASD than in unmatched TD children (mean (95% CI) for ASD = 1.19 μg/g creatinine (-0.79, 3.17) vs. TD = 4.63 μg/g creatinine (0.80, 8.46), *p* < 0.01) [13]. Similarly, in a study from Malaysia, Wahil et al. compared 81 ASD cases and 74 TD control children (3–6 years old) and reported significantly lower GM urinary Pb levels in ASD cases compared to unmatched TD controls (ASD mean (SD) = 0.26 μg/dL (0.31), TD mean (SD) = 0.58 μg/dL (0.41), *p* < 0.01) [16]. In another case–control study in Pakistan (age- and sex-matched), Rahbar et al. compared 30 ASD cases and 30 TD controls (2–12 years old) and reported significantly lower GM BPbC in children with ASD compared to their age- and sex-matched TD controls (GM for ASD = 6.37 µg/dL vs. TD = GM 7.68 µg/dL, *p* = 0.05) [56]. However, as we mentioned earlier, there are also studies reporting contrasting findings, including higher Pb levels in children with ASD than in TD children as measured in various biological samples such as hair [9], nails [11], and red blood cells [10], as well as no association between BPbCs and ASD that was reported in the CHARGE study [14]. In addition to differences in the design and population and level of controlling for potential confounding variables such as food consumption that were mentioned earlier, a possible explanation for the inconsistent findings could be variation in biomarkers used for assessment of Pb exposure in these studies. Various specimens, including blood, urine, hair, teeth, nails, and bone, can be used to assess Pb exposure in humans. Studies have reported that while Pb levels in tooth dentin and enamel and bone are considered biomarkers of cumulative Pb exposure over a long term, BPbC represents more recent exposure [57]. BPbC is the most commonly used biomarker for Pb exposure [50,58]. Alternative biomarkers for monitoring exposure to Pb have been used by several studies in the literature; however, it is still unclear whether alternative biomarkers are superior to BPbCs as indicators of Pb exposure. Currently, BPbC measurements are considered as the most reliable recent Pb exposure indicator; however, repeated measurements may be needed for assessment of fluctuations in Pb exposure over time [59]. The lower BPbCs in ASD cases compared to TD controls that we found in our study, which were also reported in several previous studies, could possibly be explained by various behavioral and eating habits of children with and without ASD that may result in lower exposure to Pb in children with ASD than in TD children. For example, there is well-established evidence of atypical food selectivity in children with ASD [60,61,62]. We have also reported in this study that ASD cases had significantly lower consumption of various types of fruits and vegetables as well as seafood that are potential dietary sources of Pb exposure in children. Although we accounted for the possible confounding effect of dietary exposures in our multivariable analyses, understanding the biological mechanisms that can explain the lower BPBCs in ASD cases than in TD children requires further investigation. 

Although to our knowledge, this study is the first that investigated possible interactive associations of GST genes (*GSTP1*, *GSTM1*, and *GSTT1*) and ASD status in relation to BPbCs, our findings suggest associations of certain GST genotypes with significantly lower BPbCs in children with ASD than in TD controls that are in line with the literature, indicating a possible role of GST genes in detoxification of Pb [10,27,29,63,64,65] and/or their associations with ASD status [14,16]. For example, our findings show significant associations between the presence of at least one Val allele for the *GSTP1* Ile105Val polymorphism and lower BPbCs in children with ASD than in TD controls, suggesting a possible role for the Val allele in detoxification of Pb. Although knowledge about the possible biological role of different *GSTP1* alleles in detoxification of Pb is very limited, our findings are consistent with another study that investigated the association between cumulative bone Pb biomarkers and cognitive function among a Boston-based prospective cohort of men participating in the Normative Aging Study. Their findings suggested that the *GSTP1* Ile105Val polymorphism is an effect modifier for the association between Pb burden and poorer cognitive function. Specifically, they reported that among *GSTP1* Val105 variant carriers, higher tibia lead concentration was associated with decreased cognitive function measured by the Mini-Mental State Examination (MMSE) score, which was significantly stronger than the association among men with only *GSTP1* Ile alleles (interaction *p* = 0.01). They also found that the negative association between bone Pb concentration and cognitive function was stronger among participants with more *GSTP1* Val105 alleles [29]. Another study, by Yohannes et al., examined the associations between GST gene polymorphisms and blood concentrations of heavy metals including Pb in 140 Pb- and zinc (Zn)-exposed children in Kabwe, Zambia, and reported a univariable significant positive association between the *GSTT1* null genotype and BPbCs (β = 0.11, *p* = 0.02) [65]. Although in our univariable analyses we did not find associations between any of the GST genes and BPbCs, we found that among children with an insertion polymorphism (I/I or I/D genotype) for either *GSTT1* or *GSTM1*, BPbCs were significantly lower in ASD cases than in TD children, which is consistent with the previous report by Yohannes et al. [65] and suggests a possible role of the active versions of these genes in detoxification of Pb. In addition, we identified a significant gene–gene interaction (*GSTT1* and *GSTP1)* in relation to BPbCs that we accounted for in our adjusted analyses, which was in line with the combined effect of *GSTT1* and *GSTP1* genes in association with BPbC that was reported by Yohannes et al. [65]. Specifically, their findings suggest that while the combination of the *GSTT1* null genotype and the *GSTP1* Ile/Val genotype was positively associated with blood Pb concentrations (β = 0.19, *p* < 0.01), having the combination of the *GSTT1* null genotype and the *GSTP1* Ile/Ile genotype was inversely associated with BPbCs (β = −0.17, *p* = 0.03). All of these findings suggest that the genotype for GST genes, either individually or in specific combinations (gene–gene interactions), may affect the susceptibility to Pb exposure by regulating Pb detoxification. In addition, these associations also may be dependent on other oxidative stress-related conditions such as ASD status. However, replication of our findings regarding the role of GST polymorphisms in BPbCs of children with and without ASD is warranted in future studies with larger sample sizes and different populations. In addition, understanding the possible biological and behavioral mechanisms that may contribute to variation in the relationships between ASD and BPbCs by certain GST genotypes requires further investigation.

## 5. Limitations

This study has a number of limitations. First, our assessment of BPbCs did not distinguish the source of Pb exposure (organic vs. inorganic); therefore, distinct sources of Pb exposure were not discussed in detail. In addition, although we previously reported a significant correlation between cord blood and childhood BPbC in Jamaican children (Spearman r = 0.45, *p* = 0.04) that remained significant after adjusting for the child’s age and sex [66], having a single BPbC assessment at the time of enrollment limited our ability to distinguish whether the concentration was due to chronic or recent Pb exposure. Furthermore, the majority of TD controls in this study were recruited from Kingston, Jamaica, which affected the generalizability of our findings on BPbCs to all Jamaican children. In addition, the activity of GSH or GST enzyme was not measured in our study, although it may further account for the complexity in the relationship between GST genes and Pb concentrations. We also acknowledge that the significant association we found between BPbCs and the *GSTP1* rs1695 polymorphism does not directly equate to rs1695 being the true causal polymorphism. In addition, we did not make any adjustments to account for multiple comparisons. Moreover, we had limited ability to establish causal inference from our findings because of the case–control study design. Therefore, the associations reported in this study should be interpreted with caution and warrant replication in similar studies with different populations.

## 6. Conclusions

In this study, we investigated possible interactions of three GST genes (*GSTP1*, *GSTM1*, and *GSTT1*) and ASD status in relation to BPbCs in Jamaican children age 2–8 years old with and without ASD. Although we did not detect significant interactions between polymorphisms in any of the three GST genes and ASD status in relation to BPbCs, we observed significantly lower BPbCs in ASD cases than in TD controls (age- and sex-matched) only among children with certain GST genotypes, before and after adjusting for SES, parish of child’s birth, consumption of lettuce, fried plantains, and canned fish, as well as the gene–gene interaction between *GSTT1* and *GSTP1* in relation to BPbC in the adjusted models that involved these two genes. Specifically, using either the dominant or co-dominant genetic model for *GSTP1*, our findings based on both unadjusted and adjusted models suggest that while among children with the Ile/Ile genotype, there was no significant difference in the GM BPbC of ASD cases and TD controls, among children with at least one *GSTP1* Val105 allele (Val/Val or Ile/Val genotypes), ASD cases had significantly lower GM BPbC than TD children. Similarly, our findings show that among children with I* genotypes for either *GSTT1* or *GSTM1*, children with ASD had significantly lower GM BPbC than TD controls, indicating a possible role of the active enzyme encoded by *GSTT1* or *GSTM1* in detoxification of Pb before and after accounting for the covariates mentioned above. These findings suggest that the presence of at least one Val allele for the *GSTP1* Ile105Val polymorphism, as well as active variants of *GSTT1* and *GSTM1* genes, may be associated with a better Pb detoxification capacity in Jamaican children with ASD than in TD controls. Although these findings are in line with the role for GST genes in detoxification of Pb, oxidative stress, and ASD, since this is the first study to assess the interactive associations of GST genes and ASD status in relation to BPbCs, further investigation in various populations is warranted.

## Figures and Tables

**Table 1:** Comparison of ASD and TD control groups with respect to characteristics of children and their parents (344 matched pairs).

Variables		Categories	ASD Cases (*n* = 344) *n* (%)	TD Controls (*n* = 344) *n* (%)	*p* Value *
**Child**	**Sex**	Male	286 (83.1)	286 (83.1)	1.00
	**Age**	< 48 months	97 (28.2)	87 (25.3)	0.02
		≥ 48 months	247 (71.8)	257 (74.7)	
	**Race**	Afro-Caribbean	325 (94.5)	334 (97.1)	0.10
	**Place of birth** **(Parish)**	Kingston	102 (29.6)	216 (62.8)	<0.01
		Others ^a^	242 (70.4)	128 (37.2)	
**Mother’s age ^b^** **(at child’s birth)**		Age < 35 years	276 (80.5)	298 (88.2)	<0.01
		Age ≥ 35 years	67 (19.5)	40 (11.8)	
**Father’s age ^c^** **(at child’s birth)**		Age < 35 years	190 (56.2)	230 (69.7)	<0.01
		Age ≥ 35 years	148 (43.8)	100 (30.3)	
**Parental education ^d^** **(at child’s birth)**		Both up to high school ^†^	128 (37.7)	169 (51.7)	<0.01
		At least one beyond high school ^††^	210 (62.3)	158 (48.3)	
**Socioeconomic status (SES)**		Car ownership	186 (54.1)	144 (41.9)	<0.01
***GSTP1*** **^e^**		Ile/Ile	86 (25.2)	91 (26.5)	0.48
		Ile/Val	190 (55.7)	175 (51.0)	
		Val/Val	65 (19.1)	77 (22.4)	
***GSTM1*** **^f^**		DD ^g^	103 (30.3)	82 (24.0)	0.07
		I/I or I/D ^h^	237 (69.7)	259 (76.0)	
***GSTT1*** **^i^**		DD ^g^	84 (24.6)	88 (25.9)	0.60
		I/I or I/D ^h^	257 (75.4)	252 (74.1)	

* *p* values are based on Wald’s test in conditional logistic regression models. ^†^ Up to high school education refers to those who attended primary/jr. secondary, and secondary/high/technical schools. ^††^ Beyond high school education indicates those who attended a vocational or tertiary college or university. ^a^ Other parishes include all 12 parishes in Jamaica, except for Kingston parish as described previously [53]. ^b^ Mother’s age was missing for 1 ASD case and 6 TD control children. ^c^ Father’s age was missing for 6 ASD case and 14 TD control children. ^d^ Parental education was missing for 7 ASD case and 17 TD control children. ^e^ *GSTP1* genotype was missing for 3 ASD case and 1 TD control children. ^f^ *GSTM1* genotype was missing for 4 ASD case and 3 TD control children. ^g^ DD indicates the null alleles for *GSTT1* and *GSTM1*. ^h^ I/I or I/D indicate the homozygote (I/I) or a heterozygote (I/D) for *GSTT1* and *GSTM1*. ^i^ *GSTT1* was missing for 3 ASD case and 4 TD control children.

**Table 2:** Findings from conditional logistic regression (CLR) models related to associations between dietary or environmental factors and ASD case status (344 matched pairs).

Exposure Variables	Category		ASD Cases *n* (%)	TD Controls *n* (%)	MOR	95% CI	*p* Value *^b^*
**Fruit and vegetable consumption *^a^***	Root vegetables	Yam, sweet potato, or dasheen	199 (58.0)	238 (69.2)	0.59	(0.43, 0.82)	<0.01
		Carrot or pumpkin	255 (74.3)	299 (86.9)	0.44	(0.30, 0.67)	<0.01
	Leafy vegetables	Lettuce	145 (42.3)	213 (61.9)	0.39	(0.28, 0.56)	<0.01
		Callaloo, broccoli, or pak choi	240 (70.0)	280 (81.4)	0.51	(0.35, 0.75)	<0.01
		Cabbage	161 (46.9)	215 (62.5)	0.51	(0.37, 0.70)	<0.01
	Fruits	Tomatoes	190 (55.4)	255 (74.1)	0.43	(0.31, 0.61)	<0.01
		Ackee	151 (44.0)	237 (68.9)	0.30	(0.20, 0.43)	<0.01
		Avocado	128 (37.3)	208 (60.5)	0.35	(0.25, 0.50)	<0.01
		Green banana	199 (58.0)	243 (70.6)	0.55	(0.40, 0.77)	<0.01
		Fried plantains	247 (72.0)	292 (84.9)	0.48	(0.33, 0.69)	<0.01
**Seafood consumption**	Salt water fish		213 (61.9)	236 (68.6)	0.71	(0.50, 1.00)	0.05
	Fresh water fish (pond fish, tilapia)		102 (29.7)	106 (30.8)	0.94	(0.66, 1.34)	0.72
	Sardine, mackerel (canned fish)		253 (73.6)	289 (84.0)	0.52	(0.35, 0.77)	<0.01
	Tuna (canned fish)		103 (29.9)	121 (35.2)	0.78	(0.56, 1.08)	0.13
	Salted fish (pickled mackerel)		220 (64.0)	271 (78.8)	0.50	(0.36, 0.70)	<0.01
	Shellfish (lobsters, crabs)		17 (4.9)	47 (13.7)	0.33	(0.19, 0.60)	<0.01
	Shrimp		34 (9.9)	60 (17.4)	0.54	(0.34, 0.84)	0.01

*^a^* For all variables under fruit and vegetable consumption, data were missing for one ASD case; ^*b*^ *p* values are based on Wald’s test in the conditional logistic regression models.

**Table 3:** Findings from univariable general linear models that assess associations of various independent variables with blood Pb concentrations (344 matched pairs) of Jamaican children (total *n* = 688).

Variables		Category		Yes		No		*p* Value **
				Mean Pb * (μg/dL)	*N*	Mean Pb * (μg/dL)	*N*	
**Child**	**ASD status**	ASD		1.74	344	2.27	344	<0.01
	**Age**	Age > 48 (months)		2.29	504	1.35	184	0.02
	**Sex**	Male		1.99	572	1.99	116	0.97
	**Place of birth (Parish)**	Kingston		2.39	318	1.70	370	<0.01
**Socioeconomic status (SES)**		Own a car		1.72	330	2.28	358	<0.01
**Mother’s age (at child’s birth) *^a^***		≥35 years		1.73	107	2.04	574	0.08
**Parental education levels** **(at child’s birth) *^b^***		At least one of the two parents had education beyond high school		1.75	368	2.26	296	<0.01
**Fruit and vegetable consumption *^c^***		Root vegetables	Yam, sweet potato, or dasheen	2.11	437	1.79	250	0.03
			Carrot or pumpkin	2.07	554	1.66	133	0.01
		Leafy vegetables	Lettuce	2.02	358	1.95	329	0.65
			Callaloo, broccoli, or pak choi	2.06	520	1.77	167	0.07
			Cabbage	2.06	376	1.90	311	0.29
		Fruits	Tomatoes	2.16	445	1.71	242	<0.01
			Ackee	2.26	388	1.68	299	<0.01
			Avocado	2.20	336	1.80	351	<0.01
			Green banana	2.10	442	1.80	245	0.05
			Fried plantains	2.09	539	1.65	148	<0.01
**Seafood consumption**		Salt water fish		2.10	449	1.80	239	0.05
		Fresh water fish (pond fish, tilapia)		2.28	208	1.88	480	0.02
		Sardine, mackerel (canned fish)		2.13	542	1.55	146	<0.01
		Tuna (canned fish)		2.01	224	1.98	464	0.83
		Salted fish (pickled mackerel)		2.14	491	1.66	197	<0.01
		Shellfish (lobsters, crabs)		2.69	64	1.93	624	0.01
		Shrimp		2.18	94	1.96	594	0.29
**Genes** ***GSTT1 (n = 337 pairs)*** ***GSTM1 (n = 337 pairs)*** ***GSTP1 (n = 340 pairs)***		*GSTT1* (I *) *^d^*		1.96	509	2.07	172	0.52
		*GSTM1* (I *) *^d^*		2.03	496	1.90	185	0.40
		*GSTP1* (Ile/Ile)		1.96	177	2.00	511	0.77
		*GSTP1* (Val/Val)		2.00	365	1.98	323	0.87
		*GSTP1* (Ile/Val)		2.02	142	1.98	546	0.85

* Mean Pb indicates the geometric mean of Pb = Exp. [Mean (ln Pb)]; ** *p* values are based on GLMs that compare geometric mean blood Pb concentrations between children in the two groups; ^*a*^ Mother’s age was missing for 7 participants; *^b^* Parental education level was missing for 24 participants; *^c^* Fruit and vegetable consumption was missing for one participant; *^d^* I* indicates the homozygote (I/I) or a heterozygote (I/D) for *GSTT1* and *GSTM1*.

**Table 4:** Unadjusted and adjusted geometric mean blood Pb concentration (GM BPbC) by GST genotypes based on general linear models (GLMs) that include interaction between GST genes and ASD case status (ASD and TD control) *.

Models	Gene	(Column A) Genotypes Compared	Referent Genotypes	Group	Unadjusted GM BPbC (μg/dL) *^a^*			Adjusted GM BPbC (μg/dL) *^b^*		
					GM BPbC with Genotypes in Column A *^c^*	GM BPbC with Referent Genotypes *^c^*	*p* Value *^d^*	GM BPbC with Genotypes in Column A *^c^*	GM BPbC with Referent Genotypes *^c^*	*p* Value *^d^*
Co-dominant *^e^* ^†^	*GSTP1*	Ile/Ile	Ile/Val	TD Control	2.14	2.31	0.51	1.78	2.13	0.16
	*GSTP1*	Ile/Ile	Ile/Val	ASD Case	1.74	1.78	0.87	1.55	1.78	0.25
	*GSTP1*	Ile/Ile	Val/Val	TD Control	2.14	2.30	0.61	1.78	1.94	0.56
	*GSTP1*	Ile/Ile	Val/Val	ASD Case	1.74	1.68	0.80	1.55	1.50	0.82
	*GSTP1*	Ile/Val	Val/Val	TD Control	2.31	2.30	0.96	2.13	1.94	0.45
	*GSTP1*	Ile/Val	Val/Val	ASD Case	1.78	1.68	0.67	1.78	1.50	0.19
Dominant *^f^* ^††^	*GSTP1*DOM	Ile/Val or Val/Val	Ile/Ile	TD Control	2.31	2.14	0.50	2.05	1.79	0.26
	*GSTP1*DOM	Ile/Val or Val/Val	Ile/Ile	ASD Case	1.75	1.74	0.96	1.71	1.55	0.39
Recessive *^g^* ^†††^	*GSTP1*REC	Val/Val	Ile/Ile or Ile/Val	TD Control	2.30	2.25	0.86	1.92	2.02	0.67
	*GSTP1*REC	Val/Val	Ile/Ile or Ile/Val	ASD Case	1.68	1.76	0.71	1.49	1.72	0.26
Recessive ^⸸^	*GSTT1*	I/I or I/D	DD	TD Control	2.26	2.29	0.89	1.91	1.99	0.71
		I/I or I/D	DD	ASD Case	1.72	1.84	0.54	1.61	1.60	0.97
Recessive ^‡^	*GSTM1*	I/I or I/D	DD	TD Control	2.26	2.31	0.84	2.04	1.81	0.34
		I/I or I/D	DD	ASD Case	1.78	1.67	0.54	1.71	1.58	0.48

* Results are based on number of pairs reported in Table 3 for *GSTP1, GSTM1*, and *GSTT1*. GM BPbC: geometric mean blood Pb concentration. *^a^* In the unadjusted GLMs, the independent variables include pairs, ASD status, *GST* gene, and *GST* gene interaction with ASD status; *^b^* In multivariable GLMs in addition to the variables in the unadjusted model, we adjusted for place of child’s birth (parish), SES (car ownership by the family), and consumption of lettuce, fried plantains, and sardine or mackerel fish. Additionally, we accounted for the interaction between GST genes (GSTT1*GSTP1 interaction) in relation to BPbCs in adjusted models related to *GSTP1* and *GSTT1* genes; *^c^* Mean Pb indicates the geometric mean of Pb = Exp. [Mean (ln Pb)]; ^*d*^ *p* values are for the comparison of mean blood Pb concentrations of children with genotypes in “Column A” compared to those with “referent genotypes”, stratified by ASD case status (ASD and TD control), based on CONTRAST statement in the SAS program for GLMs as described in the Methods section; *^e^ GSTP1* in the co-dominant model has three categories (Ile/Ile, Ile/Val, and Val/Val); *^f^ GSTP1* (DOM) = *GSTP1* in the dominant model has two categories (Val/Val or Ile/Val, Ile/Ile); *^g^ GSTP1* (REC) = *GSTP1* in the recessive model has two categories (Val/Val, Ile/Ile or Ile/Val). ^†^ Overall interaction *p* value = 0.86 and 0.81 for unadjusted and adjusted models, respectively. ^††^ Overall interaction *p* value = 0.66 and 0.83 for unadjusted and adjusted models, respectively. ^†††^ Overall interaction *p* value = 0.68 and 0.54 for unadjusted and adjusted models, respectively. ^⸸^ Overall interaction *p* value = 0.73 and 0.77 for unadjusted and adjusted models, respectively. ^‡^ Overall interaction *p* value = 0.57 and 0.82 for unadjusted and adjusted models, respectively.

**Table 5:** Unadjusted and adjusted geometric mean blood Pb concentration (GM BPbC) by ASD status (ASD and TD control) based on general linear models (GLMs) that include interaction between GST genotypes and ASD case status (ASD and TD control) *.

Gene	Models	(Column A) Group Compared	Referent Group	Genotypes	Unadjusted Model (μg/dL) *^a^*			Adjusted Model (μg/dL) *^b^*		
					GM BPbC with Group Compared in Column A *^c^*	GM BPbC with Referent Group *^c^*	*p* Value *^d^*	GM BPbC Group Compared in Column A *^c^*	GM BPbC with Referent Group *^c^*	*p* Value *^d^*
***GSTP1***	Co-dominant *^e^* ^†^	ASD Case	TD Control	Ile/Ile	1.74	2.14	0.11	1.55	1.78	0.28
		ASD Case	TD Control	Ile/Val	1.78	2.31	<0.01	1.78	2.13	0.03
		ASD Case	TD Control	Val/Val	1.68	2.30	0.02	1.50	1.94	0.06
	Dominant *^f^* ^††^	ASD Case	TD Control	Ile/Ile	1.74	2.14	0.10	1.55	1.79	0.25
		ASD Case	TD Control	Val/Val or Ile/Val	1.75	2.31	<0.01	1.71	2.05	0.01
	Recessive *^g^* ^†††^	ASD Case	TD Control	Ile/Ile or Ile/Val	1.76	2.25	<0.01	1.72	2.02	0.06
		ASD Case	TD Control	Val/Val	1.68	2.30	0.02	1.49	1.92	0.01
***GSTT1***	Recessive ^⸸^	ASD Case	TD Control	DD	1.84	2.29	0.09	1.60	1.99	0.09
		ASD Case	TD Control	I/I or I/D	1.72	2.26	<0.01	1.61	1.91	0.01
***GSTM1***	Recessive ^‡^	ASD Case	TD Control	DD	1.67	2.31	0.01	1.58	1.81	0.28
		ASD Case	TD Control	I/I or I/D	1.78	2.26	<0.01	1.71	2.04	0.01

* Results are based on number of pairs reported in Table 3 for *GSTP1, GSTM1*, and *GSTT1*. GM BPbC: geometric mean blood Pb concentration of children. *^a^* In the unadjusted GLMs, the independent variables include pairs, ASD status, *GST* gene, and *GST* gene interaction with ASD; *^b^* In multivariable GLMs, in addition to the variables in the unadjusted model, we adjusted for place of child’s birth (parish), SES (car ownership by the family), and consumption of lettuce, fried plantains, and sardine or mackerel fish. Additionally, we accounted for the interaction between GST genes (GSTT1*GSTP1 interaction) in relation to blood Pb concentrations in adjusted models related to *GSTP1* and *GSTT1* genes; *^c^* Mean Pb indicates the geometric mean of Pb = Exp. [Mean (ln Pb)]; ^*d*^ *p* values are for the comparison of mean blood Pb concentrations of children with ASD case status in “Column A” compared to those with TD control status in “referent group”, stratified by *GST* genotypes, based on CONTRAST statement in the SAS program for GLMs as described in the Methods section; *^e^ GSTP1* in the co-dominant model has three categories (Ile/Ile, Ile/Val, and Val/Val); *^f^ GSTP1* (DOM) = *GSTP1* in the dominant model has two categories (Val/Val or Ile/Val, Ile/Ile); *^g^ GSTP1* (REC) = *GSTP1* in the recessive model has two categories (Val/Val, Ile/Ile or Ile/Val). ^†^ Overall interaction *p* value = 0.86 and 0.81 for unadjusted and adjusted models, respectively. ^††^ Overall interaction *p* value = 0.66 and 0.83 for unadjusted and adjusted models, respectively. ^†††^ Overall interaction *p* value = 0.68 and 0.54 for unadjusted and adjusted models, respectively. ^⸸^ Overall interaction *p* value = 0.73 and 0.77 for unadjusted and adjusted models, respectively. ^‡^ Overall interaction *p* value = 0.57 and 0.82 for unadjusted and adjusted models, respectively.

## Data Availability

The data analyzed in this study are from two grants (i.e., R21 and R01). The data from R01 are or will be publicly available through the National Database for Autism Research (NDAR). Data from R21 will also be available upon request from the corresponding author.
